# Return Period Evaluation of the Largest Possible Earthquake Magnitudes in Mainland China Based on Extreme Value Theory

**DOI:** 10.3390/s21103519

**Published:** 2021-05-18

**Authors:** Ning Ma, Yanbing Bai, Shengwang Meng

**Affiliations:** Center for Applied Statistics, School of Statistics, Renmin University of China, Beijing 100872, China; ning.ma@ruc.edu.cn (N.M.); mengshw@ruc.edu.cn (S.M.)

**Keywords:** seismic hazard, extreme value theory, return level, right truncation

## Abstract

The largest possible earthquake magnitude based on geographical characteristics for a selected return period is required in earthquake engineering, disaster management, and insurance. Ground-based observations combined with statistical analyses may offer new insights into earthquake prediction. In this study, to investigate the seismic characteristics of different geographical regions in detail, clustering was used to provide earthquake zoning for Mainland China based on the geographical features of earthquake events. In combination with geospatial methods, statistical extreme value models and the right-truncated Gutenberg–Richter model were used to analyze the earthquake magnitudes of Mainland China under both clustering and non-clustering. The results demonstrate that the right-truncated peaks-over-threshold model is the relatively optimal statistical model compared with classical extreme value theory models, the estimated return level of which is very close to that of the geographical-based right-truncated Gutenberg–Richter model. Such statistical models can provide a quantitative analysis of the probability of future earthquake risks in China, and geographical information can be integrated to locate the earthquake risk accurately.

## 1. Introduction

Earthquake disasters have resulted in substantial economic losses in recent years. Thus, the effective management of the risk of seismic hazards has become crucial [1]. Although pervasive uncertainties exist in the seismic hazard process, potential shaking should be estimated for social decision making [2,3]. Probabilistic seismic hazard analysis (PSHA) can provide necessary information, to the prevention of earthquake damages [4,5]. Therefore, it is vital to establish a suitable model for describing and predicting the risk of earthquakes accurately.

In seismology, earthquake forecasting models are divided into three categories. The first is the statistical probability prediction model, which is based on the Gutenberg–Richter (GR) relation [6]. The GR relation forms the foundation of statistical probability prediction of earthquakes, and many earthquake forecasting models have been derived on this basis. Such models include modified GR model [7], the high-resolution time-independent grid-based model [8], the asperity-based likelihood model [9], and entropy of earthquakes model [10]. The second category is physical prediction models, which are divided into two types. One is based on the complex observable space–time patterns of earthquake behavior. The basic assumption is that future earthquakes are more likely to occur in areas where earthquakes have occurred in the past. These approaches include the RI method [11], PPE model [12], PI method [13], and RELM model [14]. The other is based on seismic quiescence phenomena prior to large earthquakes [15], such as the RTM algorithm [16] and M8 algorithm [17,18]. The third category is hybrid forecasting models, in which different types and principles of forecasting models are combined to utilize their respective advantages and to improve the forecasting performance. These are models that mix statistical probability forecasting models with physical earthquake forecasting models, such as the ETAS fault model [19,20] and CRS-unc model [21].

High requirements exist for the geographical information of the region and the geographical data of earthquakes when using PSHA models to predict earthquakes. A PSHA model is based on understanding the mechanism of earthquakes in depth through modeling to predict the occurrence of earthquakes in the future [2]. This method is more suitable for analyzing a region in combination with local geographical characteristics. From a statistical model perspective, directly obtained repeatable multisensor observation data are more suitable for areas with complex geographical features and large regions for analysis. Statistical analysis methods can analyze and classify the characteristics of regional seismic events according to the physical characteristics of the earthquake. In combination with a statistical model, the earthquake prediction results can subsequently be provided from a data-driven perspective.

Extreme value theory deals with the statistical laws of extreme values of a random variable and is dedicated to the statistical analysis of rare events. This approach can effectively describe the tail characteristics of the data and involves simple calculations. Extreme value theory is an indispensable tool in the study of natural disasters. It is a mainstream model that fits the tail distribution of catastrophe risk data, and it is used extensively in various fields, such as hydrology, meteorology, and earth science [22,23,24,25]. Fréchet, Fisher, and Tippett first used a statistical model to describe the behavior of the maximum and minimum values of a random variable, and they proposed the extreme value theorem, noting that the maximum or minimum value fits three-parameter distributions [26,27]. Gnedenko presented proof of the extreme value theorem [28], which became widely used in the application field. The peaks-over-threshold (POT) method determines the probability distribution of extreme events that exceed a threshold. In the seismic area, the modeling of extreme values and the estimation of tail parameters have been investigated via the POT method [29,30,31]. Several studies have revealed that extreme value theory offers valuable properties in describing the characteristics of the right tail of earthquake magnitude data compared to other statistical models [32,33,34]. The modeling of extreme events has received extensive attention in recent years. Estimating the risk of rare occasions by in turn estimating the quantile of the extreme events or the corresponding return period is crucial [35,36,37]. Future earthquake catastrophe events can be predicted through the extreme values that are obtained by fitting models, and the risk of rare events can be evaluated by estimating the high quantile or the corresponding return period.

Although extreme value theory has undergone extensive development since its initial presentation, certain controversial aspects remain. One of these issues is that when the shape parameter of the extreme value theory model is ξ≥0, the right tail tends to infinity, which will cause irrationality for several application scenarios. For example, the earthquake magnitude cannot tend to infinity. The manner in which to add an appropriate right truncation point to the model has been studied further. In the existing research, two types of models that include the right truncation point are available. One is the provision of the right truncation point using a probability and statistics method in addition to the POT method [38]. The other is based on the right-truncated GR distribution [39,40,41], which is obtained by the geographical characteristics of earthquake magnitude data. Researchers have solved the problem of using unbounded probability mass resulting in unreasonably large or physically impossible levels. All these studies considered the situation in which ξ>0 [42,43,44]. Beirlant observed that the above extreme value method could not capture truncation at high levels, even when using a negative extreme value index [38]. However, in several other fields, such as hydrology and earthquake magnitude modeling, the underlying distribution appears to be lighter than the Pareto tail. Thus, he proposed a method to adapt to the truncation in the maximum convergence range. This right truncation model is suitable for ξ>−0.5 [38]. The GR model based on empirical evidence was proposed in 1956. The original GR model does not have an upper limit. Right truncation was subsequently introduced into the model, which assumed that the earthquake magnitude followed a double truncated exponential distribution [40]. The GR model can provide a different view of the statistical analysis of earthquake magnitudes from the perspective of geophysics, based on which the estimation for the largest expected earthquake in a given future time interval can be obtained appropriately [45].

China is a country with a high incidence of earthquakes, and it is located between two major seismic belts: the Pacific Rim and Eurasian seismic belts. The seismic fault zone, which is compressed by the Pacific plate, Indian plate, and Philippine Sea plate, is very active, resulting in large earthquake intensities, a wide distribution range, and a high disaster rate, particularly for Mainland China. Moreover, Mainland China has a large geographical area and a complex geographical environment. Different regions have different geographical characteristics and their historical seismic data vary substantially [46,47]. Thus, it is necessary to study the magnitudes of earthquakes in Mainland China. Cluster analysis can be used to explore the underlying structures in data, and it is a useful technique for discovering and extracting information that may not have been noticed before. Cluster analysis can divide data into several categories according to the data characteristics, making the similarity among objects in the same category stronger than the similarity among objects in different categories [48]. Therefore, more detailed and targeted research results can be obtained by creating a cluster according to the characteristics of historical seismic records and subsequently analyzing the clustered regions. Cluster analysis can be used to provide earthquake zoning for Mainland China scientifically based on the geographical features of the earthquakes, and the characteristics of the earthquakes in each earthquake zone can be reasonably summarized. China has a long history of earthquake recording. The dataset from the China Earthquake Networks Center provides ground-based observations of historical earthquakes. According to the cluster analysis results of the historical earthquakes of Mainland China earthquakes, in combination with map visualization, we can further analyze the regional characteristics of earthquakes in Mainland China. Current cluster analysis methods include hierarchical clustering [49], K-means clustering [50], two-stage clustering, fuzzy c-means [51], partitioning around medoids [52], hidden Markov models [53], and mixture models [54]. Hidden Markov models and mixture models require large datasets, whereas fuzzy c-means and hierarchical clustering have high complexity. Among these methods, K-means clustering has been used extensively owing to its low complexity and ease of implementation.

In this study, the magnitude of the possible largest earthquake in Mainland China was analyzed through parametric models in combination with geospatial information under both clustering and non-clustering. The probability statistical model based on the GR relationship from the perspective of seismology was used as a benchmark, and the extreme value statistical model was analyzed from a statistical perspective. In this study, statistical models were used to determine the probability of the occurrence of earthquake risk from a probability perspective. The estimation of the return level of the earthquake magnitude and the analysis of the maximum possible magnitude can provide a valuable reference for the prevention and emergency response of future earthquakes in China. This work did not infer the occurrence of an earthquake, nor did it conduct research on the cause of an earthquake. Several natural disaster events have statistical periodicity [55] and not every natural disaster event is isolated [56]. Through this research, we hope to discover certain statistical laws of extreme seismic events, which may be used as a supplement to previous studies based on geological structures. The statistical extreme value model can quantify and predict the occurrence probability of earthquake magnitudes in the future, and it offers superior prediction performance and wider applicability compared to seismology models based on earthquake occurrence mechanisms.

The remainder of this paper is organized as follows: Section 2 introduces the statistical methods used to study earthquakes in Mainland China. Section 3 introduces the data used in this study and presents a discussion on the obtained model and results. Finally, Section 4 provides the conclusions.

## 2. Materials and Methods

Achieving high-precision prediction of earthquakes is difficult owing to their complex mechanisms and geographical diversity. However, the occurrence of earthquakes is not completely disordered, and to a certain extent, probability predictions can be performed based on statistical parameter models. The right-truncated GR model assumes that the earthquake magnitude follows a double-truncated exponential distribution, and this model can provide a statistical analysis of the earthquake magnitude from a geophysical perspective. The GR relationship, which has been proven by theory, experience, and empirical studies in seismology, provides a reliable model for the earthquake magnitude distribution. In this study, we assume that the right-truncated GR model represents the geophysical understanding of the magnitude distribution of earthquakes. Statistical models of extreme value offer a wide range of applications for assessing the risk of rare events, such as catastrophic floods, huge losses of insurance companies, the occurrence of financial risks, and forest fires. In this study, we attempted to compare the estimation effects of extreme value statistical models and used the right-truncated GR model as a benchmark for comparing the estimation results.

This section introduces right-truncated GR model and the distribution of extreme value statistical models. Moreover, the analytical methods used in this study are presented.

### 2.1. Right-Truncated GR Distribution

In the field of seismology, several models that are suitable for different application scenarios have been developed from the statistical probability prediction model based on the GR relationship. Among these, the right-truncated GR model can provide a reasonable distribution approximation and an appropriate upper limit for the earthquake magnitude. If the earthquake magnitude distribution function is F(x), the largest observed magnitude $M_{n}=\max\left(X_{1},X_{2},\cdots ,X_{n}\right)$ has the following cumulative distribution function:

(1) $$F_{M_{n}}\left(m\right)=\left\{\begin{array}{cc} 0 & \;\mathrm{for}\;\;m<t_{m}\\ {\left[F_{X}\left(m\right)\right]}^{n} & \;\mathrm{for}\;\;t_{m}\leq m\leq T_{m}.\\ 1 & \;\mathrm{for}\;\;m>T_{m} \end{array}\right.$$

where $t_{m}$ is the minimum value among the observed values participating in the estimation and $T_{m}$ is the right truncation point. The expected value of $M_{n}$ is:

(2) $$E\left(M_{n}\right)=\int_{t_{m}}^{T_{m}}m\;dF_{M_{n}}\left(m\right)=T_{m}-\int_{t_{m}}^{T_{m}}F_{M_{n}}\left(m\right)dm.$$

When the expected value of the largest observed magnitude $E(M_{n})$ is replaced with the largest observed magnitude $M_{n}$, $T_{m}$ and the observed values exhibit the following relationship:

(3) $$T_{m}=M_{n}+\int_{t_{m}}^{T_{m}}{\left[F_{X}\left(m\right)\right]}^{n}\;dm.$$

The magnitude of an earthquake is derived from a double-truncated exponential distribution for the GR law:

(4) $$1-F_{X}\left(m\right)=\frac{e^{-\beta m}-e^{-\beta T_{m}}}{e^{-\beta t_{m}}-e^{-\beta T_{m}}}.$$

The estimation of the parameters can be obtained using Cramér approximation. According to Cramér approximation [57], when *n* is sufficiently large, ${\left[F_{X}\left(m\right)\right]}^{n}$ can be approximately equal to $\exp\left\{-n\left[1-F_{X}\left(m\right)\right]\right\}$. Using this replacement, Equation (3) can be solved by iteration [58]:

(5) $$T_{m}=M_{n}+\left[\frac{E_{1}\left(n_{2}\right)-E_{1}\left(n_{1}\right)}{\beta \exp(-n_{2})}+t_{m}\exp\left(-n\right)\right],$$

where

(6) $$n_{1}=\frac{n}{1-\exp(-\beta \left(T_{m}-t_{m}\right))},n_{2}=n_{1}\exp\left(-\beta \left(T_{m}-t_{m}\right)\right)$$

and $E_{1}$ are exponential integral functions, which are defined as $E_{1}\left(z\right)=\int_{z}\exp\left(-\zeta \right)/\zeta d\zeta$. The parameter β is based on the truncated GR distribution using maximum likelihood for the estimation [41,59]. It is estimated iteratively using the following equation:

(7) $$\frac{1}{\beta }=\bar{X}-t_{m}+\frac{\left(T_{m}-t_{m}\right)\exp\left(-\beta \left(T_{m}-t_{m}\right)\right)}{1-\exp(-\beta \left(T_{m}-t_{m}\right))},$$

where $\bar{X}=\sum_{i=1}^{n}X_{i}/n$ is the sample mean. Using Taylor expansion,

(8) $$\beta =\beta_{0}\left(1-\beta_{0}\frac{\left(T_{m}-t_{m}\right)\exp\left(-\beta_{0}\left(T_{m}-t_{m}\right)\right)}{1-\exp(-\beta \left(T_{m}-t_{m}\right))}\right).$$

In the above, $\beta_{0}$ = $\frac{1}{\bar{X}-t_{m}}$ is the estimate of β according to Aki-Utsu [60,61]. As this method does not use iteration, it was selected for computational simplicity.

### 2.2. Statistical Models of Extreme Values

Let $X_{1},X_{2},\cdots ,X_{n}$ be independent and identically distributed random variables with the cumulative distribution function *F* and let $M_{n}=\max\left(X_{1},X_{2},\cdots ,X_{n}\right)$ represent the maximum, which can be approximated by the generalized extreme value distribution. Suppose that there exist sequences of constants $a_{n}>0$ and $b_{n}\in R$, such that

(9) $$P(\frac{M_{n}-b_{n}}{a_{n}}\leq z)\to G(z),\;when\;n\to \infty ,$$

where G(z) is a distribution function. This theorem is an analogy to the central limit theorem, with $b_{n}$ as a location parameter and $a_{n}$ as a scale parameter. The distribution function of the standardized variable will tend to G(z) when *n* tends to infinity. Two fundamental methods for modeling the extreme values of a random variable are available: the block maxima (BM) and POT methods.

The tail characteristics of certain statistical distributions exhibit truncation effects when studying the behavior of extreme data values. Beirlant created the POT model with right truncation when the shape parameter ξ>−0.5, which can be obtained by pseudo-maximum likelihood estimation [38]. The truncation at the right tail is considered as the maximum value that may occur in the future, as predicted by the existing data.

#### 2.2.1. BM Method

The BM method estimates the probability distribution G(z). This method involves dividing an independent random observation sequence into non-overlapping intervals in terms of the time, length, or other criteria, under the premise that the overall distribution function F(x) is unknown. Thereafter, the maximum value in each interval is selected as the sample data and the generalized extreme value distribution is determined. Following the parameter estimation, the overall distribution function can be obtained. The BM method can describe the behavior of the maximum value that occurs in a cycle, which may be one day, one month, or one year. According to the extreme value theorem, when the sample size is sufficiently large, the distribution of the regional maximum tends to be a Gumbel, Fréchet, or Weibull distribution. The parameters of these distributions are the location parameter (μ), which represents the central tendency and range, the scale parameter (σ), which represents the central tendency and dispersion, and the shape parameter (ξ), which represents the degree of dispersion and higher-order moments. The following mathematical expressions describe the above three probability distributions:

Gumbel cumulative distribution:

(10) $$F\left(x\right)=e^{-e^{\frac{-(x-\mu )}{\sigma }}}$$

Density function of Gumbel:

(11) $$f\left(x\right)=\frac{e^{\frac{-(x-\mu )}{\sigma }}e^{-e^{\frac{-(x-\mu )}{\sigma }}}}{\sigma }\;\;x\in \left(-\infty ,\infty \right)$$

Fréchet cumulative distribution:

(12) $$F\left(x\right)=\left\{\begin{array}{cc} 0, & x\leq \mu \\ e^{-{\left(\frac{x-\mu }{\sigma }\right)}^{-\xi }}, & x>\mu \end{array}\right.$$

Density function of Fréchet:

(13) $$f\left(x\right)=\frac{\xi }{\sigma }{\left(\frac{x-\mu }{\sigma }\right)}^{-1-\xi }e^{-{\left(\frac{x-\mu }{\sigma }\right)}^{-\xi }},x>\mu$$

Weibull cumulative distribution:

(14) $$F\left(x\right)=\left\{\begin{array}{cc} 0, & x<\mu \\ 1-e^{-{\left(\frac{x-\mu }{\sigma }\right)}^{\xi }}, & x\geq \mu \end{array}\right.$$

Weibull density function:

(15) $$f\left(x\right)=\frac{\xi }{\sigma }{\left(\frac{x-\mu }{\sigma }\right)}^{\xi -1}e^{-{\left(\frac{x-\mu }{\sigma }\right)}^{\xi }},x\geq \mu .$$

The distributions of Gumbel, Fréchet, and Weibull can be generalized into a single family of distributions [62,63], which is known as the generalized extreme value (GEV) distribution:

(16) $$G\left(x\right)=\left\{\begin{array}{cc} e^{-{\left(1+\xi \left(\frac{x-\mu }{\sigma }\right)\right)}^{(}-\frac{1}{\xi })}, & \xi \neq 0\\ e^{-e^{\frac{-(x-\mu )}{\sigma }}}, & \xi =0 \end{array}\right.$$

If ξ > 0, the domain is [μ−σ/ξ,+∞). If ξ < 0, the domain is (−∞,μ−σ/ξ]. If ξ=0, the domain is x∈(−∞,+∞).

The value of the shape parameter (ξ) controls the approximation of the limit distribution. If ξ>0, the Fréchet distribution is used, when ξ = 0, the Gumbel distribution is selected, and if ξ<0, the Weibull distribution is used. When using the BM method to divide the interval, the sequence will be close to independence and autocorrelation may be omitted if the interval is sufficiently large. However, some significant variability may not be detected.

#### 2.2.2. POT Method

The goal of this method is to determine the probability distribution of extreme values that exceed the threshold. Let $X_{1},X_{2},\cdots ,X_{n}$ be independent and identically distributed random variables with a cumulative distribution function *F*. Select a sufficiently large number *u* as a fixed threshold. All variables greater than *u* are extreme variables. If $X_{i}-u\geq$ 0, $Y_{i}=X_{i}-u$ is known as exceedance. The function of excesses over a threshold is given as follows:

(17) $$F_{u}\left(x\right)=P\left(X-u\leq x|X>u\right)=\frac{F(x+u)-F(u)}{1-F(u)}$$

This method is optimal when the threshold is sufficiently high, with a large number of observations, which can be explained by the Pickands theory [64]. The selection of the threshold is key to the POT. If the threshold is too low, there will be more observations, but the prediction will be biased; if the threshold is too high, there will be fewer observations, which will lead to greater variance in the parameter estimation.

Current threshold selection methods include graphical diagnostics, heuristic methods, and automatic threshold selection. Graphical diagnostics are traditional methods that visually select the threshold through images, which mainly include the mean residual life (MRL) plot and Hill plot [29]. Heuristic methods include the upper 10% rule, the square root rule, and the empirical rule [65]. Although these methods have no theoretical basis, they are easy to calculate. Automatic threshold selection includes a shape parameter stability test using the likelihood ratio test and score test [66], threshold selection based on bootstrap [67], and a bias reduction procedure [68]. However, an excessively high threshold tends to be selected in heuristic methods and automatic threshold selection, which leads to unstable parameter estimation. Therefore, this study uses the most common graphical diagnostics method for the threshold selection.

The MRL plot and Hill plot are used to determine the threshold. In the MRL plot, the excess mean of random variables is calculated under different thresholds, the linearity of which can be the reference for selecting the threshold. This threshold selection method is dependent on the subjective criteria of the researcher. The Hill plot method uses the order statistic $X_{\left(1\right)}\leq X_{\left(2\right)}\leq \cdots \leq X_{\left(n\right)}$ that corresponds to the independent and identically distributed random variable $X_{1},X_{2},\cdots ,X_{n}$ to construct the Hill statistic $H_{K}$:

(18) $$H_{k}=\frac{1}{k}\sum_{j=1}^{k}\left(\ln X_{\left(j\right)}-\ln X_{\left(k+1\right)}\right)$$

Use *k* as the horizontal axis and ${H_{u}}^{-1}$ as the vertical axis of the plot and select the sample point corresponding to the abscissa *k* of the stable starting point of $H_{k}$ in the Hill plot as the threshold.

The Pickands theory [64] states that the limit distribution for excesses over the threshold ($F_{u}\left(x\right)$) can be approximated effectively by the generalized Pareto (GP) distribution, which uses three parameters: the location parameter (μ), scale parameter (σ), and shape parameter (ξ). The cumulative distribution of the GP is expressed in the following form:

(19) $$F\left(x\right)=\left\{\begin{array}{cc} 1-{\left(1+\frac{\xi (x-\mu )}{\sigma }\right)}^{-\frac{1}{\xi }}, & \xi \neq 0\\ 1-e^{-(\frac{x-\mu }{\sigma })}, & \xi =0 \end{array}\right.$$

If ξ≥ 0, the domain is x≥μ. If ξ< 0, the domain is $\mu \leq x\leq \mu -\frac{\sigma }{\xi }$. If ξ=0, the distribution is an exponential distribution.

The density function of the GP is determined as follows:

(20) $$f\left(x\right)=\left\{\begin{array}{cc} \frac{1}{\sigma }{\left(1+\frac{\xi (x-\mu )}{\sigma }\right)}^{(}-\frac{1}{\xi }-1), & if\;\xi \neq 0\\ \frac{e^{-(\frac{x-\mu }{\sigma })}}{\sigma }, & if\;\xi =0 \end{array}\right.$$

The model parameter estimation is obtained by maximum likelihood estimation. These estimators exhibit asymptotic properties, such as consistency, normality, and validity [69].

#### 2.2.3. Right-Truncated POT Distribution

The POT model with right truncation under the shape parameter ξ> −0.5 is obtained by the pseudo-maximum likelihood estimation method. The parameter estimation method and the estimation formula for the right truncation point are provided in [38].

Suppose that the order statistic $X_{\left(1\right)}\leq X_{\left(2\right)}\leq \cdots \leq X_{\left(n\right)}$ corresponds to the random variable $X_{1},X_{2},\cdots ,X_{n}$, the threshold value in the POT model is $X_{\left(n-k\right)}$, and the excesses are defined as $E_{j,k}=X_{\left(n-j+1\right)}-X_{\left(n-k\right)}$, where $E_{1,k}=X_{\left(n\right)}-X_{\left(n-k\right)}$.

The log maximum likelihood function of the parameters and the estimation formula of the right truncation point are as follows [38]:

When ξ = 0, the log maximum likelihood function is expressed as follows:

(21) $$\log L_{k,n}\left(\sigma \right)=-\left(k-1\right)\log\sigma -\sum_{j=2}^{k}\frac{E_{j,k}}{\sigma }-\left(k-1\right)\log\left(1-\exp\left(-\frac{E_{1,k}}{\sigma }\right)\right)$$

with the estimation of the right truncation point and the estimation of the quantile:

(22) $${\hat{T}}_{m}=X_{\left(n-k\right)}+\sigma \log\left[1+k\frac{\exp\left(\frac{E_{1,k}}{\sigma }\right)-1}{k-\exp\left(\frac{E_{1,k}}{\sigma }\right)}\right]$$

(23) $${\hat{Q}}_{Y}\left(1-p\right)=X_{\left(n-k\right)}+\sigma \log\left[\frac{{\hat{D}}_{T,k}+1}{{\hat{D}}_{T,k}+p}\right],$$

where ${\hat{D}}_{T,k}=\max\left[0,\frac{1}{k}\frac{k-\exp\left(\frac{E_{1,k}}{\sigma }\right)}{\exp\left(\frac{E_{1,k}}{\sigma }\right)-1}\right]$.

When ξ≠0, let τ=ξ/σ; then, the log maximum likelihood function is expressed as follows:

(24) $$\begin{array}{cc} \log L_{k,n}\left(\xi ,\tau \right)= & \left(k-1\right)\log\tau -\left(k-1\right)\log\xi -\left(1+\frac{1}{\xi }\right)\sum_{j=2}^{k}\log\left(1+\tau E_{j,k}\right)\\ \; & -\left(k-1\right)\log\left(1-{\left(1+\tau E_{1,k}\right)}^{-1/\xi }\right). \end{array}$$

By deriving the parameter (ξ,τ) separately, the parameter values can be solved using the following equations:

(25) $$\left\{\begin{array}{c} \frac{1}{k-1}\sum_{j=2}^{k}\log(1+\hat{\tau }E_{j,k})+\frac{{\left(1+\hat{\tau }E_{1,k}\right)}^{-1/\hat{\xi }}\log\left(1+\hat{\tau }E_{1,k}\right)}{1-{\left(1+\hat{\tau }E_{1,k}\right)}^{-1/\hat{\xi }}}=\hat{\xi }\\ \frac{1}{k-1}\sum_{j=2}^{k}\frac{1}{1+\hat{\tau }E_{j,k}}=\frac{1}{1+\hat{\xi }}\frac{1-{\left(1+\hat{\tau }E_{1,k}\right)}^{-1-1/\hat{\xi }}}{1-{\left(1+\hat{\tau }E_{1,k}\right)}^{-1/\xi }} \end{array}\right.$$

with the estimation of the right truncation point and estimation of quantile:

(26) $${\hat{T}}_{m}=X_{\left(n-k\right)}+\frac{1}{\tau }\left[{\left(\frac{1-\frac{1}{k}}{{\left(1+\tau \left(X_{\left(n\right)}-X_{\left(n-k\right)}\right)\right)}^{-\frac{1}{\xi }}-\frac{1}{k}}\right)}^{\xi }-1\right]$$

(27) $$\hat{Q}\left(1-p\right)=X_{\left(n-k\right)}+\frac{1}{{\hat{\tau }}_{k}}\left[{\left\{\frac{{\hat{D}}_{T,k}+1}{{\hat{D}}_{T,k}+p}\right\}}^{{\hat{\xi }}_{k}}-1\right],$$

where ${\hat{D}}_{T,k}=\max\left\{0,\frac{{\left(1+{\hat{\tau }}_{k}E_{1,k}\right)}^{-1/{\hat{\xi }}_{k}}-\frac{1}{k}}{1-{\left(1+{\hat{\tau }}_{k}E_{1,k}\right)}^{-1/\xi_{k}}}\right\}$.

### 2.3. Model Selection

The model selection method needs to determine whether the shape parameter ξ of statistical models of extreme values is zero. Numerous methods are available for model selection. In this study, we use the likelihood ratio test, Akaike Information Criterion (AIC), and Bayesian Information Criterion (BIC) to determine whether ξ is zero and compare the goodness of fit of the models.

#### 2.3.1. Likelihood Ratio Test

The likelihood ratio test is a hypothesis test that enables the comparison of two models: the model corresponding to the null hypothesis has *p* parameters, whereas the model corresponding to the alternative hypothesis has p+1 parameters. In this study, the likelihood ratio test is used to test whether the shape parameter (ξ) of the GEV model or POT model is zero. Suppose that the original hypothesis is that the shape parameter (ξ) is equal to zero and the alternative hypothesis is that the shape parameter (ξ) is not equal to zero. If the null hypothesis is not rejected (*p*-value > 0.05) and the shape parameter (ξ) is not significant, the Gumbel distribution or exponential distribution is used. The statistic of the likelihood ratio test in this study approximately obeys the chi-square distribution with one degree of freedom.

#### 2.3.2. Goodness of Fit

AIC and BIC measure the goodness of fit of a statistical model. An optimal model can be selected by comparing the AIC and BIC values of a set of models. Suppose that *k* is the number of parameters, *L* is the log maximum likelihood estimation function, and *n* is the sample size. The formulae for AIC and BIC are as follows:

(28) AIC=2k−2lnL

(29) BIC=−2lnL+klnn.

A higher likelihood function value indicates a better fit of the theoretical model, corresponding to lower AIC and BIC values. In general, the lower AIC and BIC values of the model result in a superior model to be used. BIC assigns greater penalization to models than AIC, which indicates that the simplest models are preferable. GEV and GP models have up to three parameters. These two standards can aid in the model selection.

### 2.4. K-Means Clustering

The K-means clustering algorithm, which was proposed in 1956 [50], is used extensively in various fields, such as biology, psychology, and market research. Although new clustering methods have been proposed in recent decades, K-means has always been one of the most commonly used approaches [70]. The concept of the K-means algorithm is to gather each group into its nearest centroid. The process is as follows:

1. Randomly select *K* objects to form *K* initial clusters.
2. Modify each cluster and assign each sample to the cluster with the nearest mean value and recalculate the centroid of each cluster.
3. Repeatedly redistribute each cluster, until no sample enter or exit clusters remain.

To prevent the randomness of the initial centroid from causing changes in the clustering results, the K-means algorithm is run repeatedly to determine the most stable solution. As the K-means algorithm needs to identify the appropriate number of categories K, partitioning methods must be used to determine the best K value prior to clustering.

## 3. Results and Discussion

The models mentioned in Section 2 were applied to Mainland China and the zones formulated by the K-means clustering results. This was realized to obtain suitable statistical models of extreme values for modeling the earthquake magnitudes and to determine the return levels of the earthquake magnitudes within 50 years. For each return level, we used bootstrap with 5000 replicates to provide a 95% confidence interval, which can quantify the probability that the true value of the return period falls around the estimated result.

The return period and return levels are generally used to describe and quantify risk. The return level is the 100p% quantile of a variable and *p* is the probability that the variable will exceed the return level in one year; that is, $P\left(X\geq Z_{p}\right)=p$. The return period 1/p is the average time of the variable exceeding the return level for the second time.

In Section 3.4, we present the QQ plots of the right tail to compare the differences between the statistical models of extreme values and the right-truncated GR model. The QQ plots can make a detailed and intuitive comparison of the quantiles at the right end of each model, thereby further explaining the difference in the estimation of the return levels by each model.

### 3.1. Earthquake Records of Mainland China

Data from 1920 to 2020 in Mainland China were selected for analysis. The data were obtained from the Earthquake Science Data Sharing Center of China Earthquake Administration, including the time, latitude, longitude, magnitude, and depth of the earthquakes. Earthquakes with magnitudes below 5 Mw may be felt but generally do not cause damage to buildings. Earthquakes with magnitudes above 5 Mw are considered as strong earthquakes and may cause damage to buildings. Thus, earthquakes with a magnitude of 5 Mw and above were selected for analysis to offer greater practical significance and application value. The earthquakes involved in the analysis were all mainshocks, excluding foreshocks and aftershocks. A total of 907 seismic events were used, which could be considered as independent.

**Figure 1:**
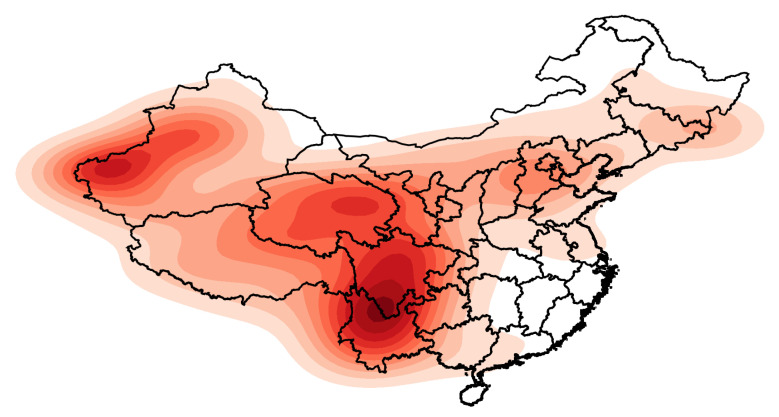
Heat map of earthquake frequency in Mainland China.

**Figure 2:**
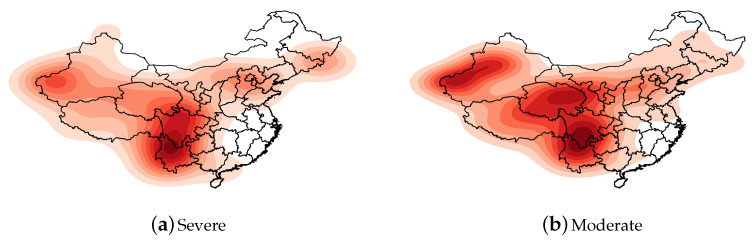
Heat maps of frequency of earthquakes in Mainland China classified by magnitude (severe: Mw ≥ 6; moderate: 6 > Mw ≥ 5).

The heat maps presented in Figure 1 and Figure 2 were drawn according to the longitude and latitude of each earthquake. The intensity of the color represents the kernel density: that is, the relative frequency of earthquakes. In Figure 2, the earthquakes are separated into two groups according to their magnitudes.

In Figure 1, the darker colors are the areas with a large number of earthquakes with magnitudes of 5 Mw and above in the history of Mainland China: the junction of Yunnan and Sichuan and the junction of Xinjiang Uygur Autonomous Region, Kyrgyzstan and Tajikistan, and Qinghai Province. Few earthquakes with magnitudes of 5 Mw and above occurred in southeastern Mainland China from 1920 to 2020, which led to the kernel density approaching zero. Moreover, there were two relatively concentrated areas of earthquakes in the central part of Hebei Province and the junction of Jilin and Heilongjiang.

Earthquakes with a magnitude of 6 Mw and above are violent earthquakes that may have serious effects on buildings and human life. It is necessary to investigate the geospatial distribution characteristics of such destructive earthquakes. Therefore, we divided the earthquakes with magnitudes of 5 Mw and above into two levels according to the magnitude: severe (Mw ≥ 6) and moderate (5 ≤ Mw < 6). A heat map of the frequency of earthquakes for each level was drawn, as indicated in Figure 2.

Severe earthquakes occurred on the border of the Xinjiang Uygur Autonomous Region and the junction of Yunnan and Sichuan. Among these, the junction of Yunnan and Sichuan was the darkest, indicating that the frequency of severe earthquakes was the highest. Furthermore, severe earthquakes occurred in the Beijing–Tianjin–Hebei region and at the border of Jilin Province and Heilongjiang Province, indicating that these two regions are relatively important areas for earthquakes in northeastern China.

Moderate earthquakes occurred in three regions: the border of the Xinjiang Uyghur Autonomous Region, Qinghai Province, and the border of Yunnan and Sichuan. The Uyghur Autonomous Region has a wider range of moderate earthquakes than severe earthquakes. In Figure 2, the seismic frequency of Qinghai Province is relatively high in the heat map of the moderate earthquakes, but it is not a prominent dark spot in the heat map of severe earthquakes, which indicates that the seismic intensity of Qinghai Province was mainly on the moderate level. The relative seismic frequency at the junction of Sichuan and Yunnan is extremely prominent below the two levels, indicating that this area was the hardest hit by earthquakes in China.

### 3.2. Application of Parameter Models for Earthquake Magnitude in Mainland China

First, the BM method was applied to the maximum magnitudes per year in Mainland China. Table 1 lists the estimated parameters, estimated standard errors, and AIC and BIC values of the GEV and Gumbel models that were obtained through maximum likelihood estimation.

As the shape parameter estimated by the GEV model was less than zero and the estimated standard error was small, the shape parameter should be non-zero. The likelihood ratio test was conducted to determine whether the shape parameter was zero. A comparison of AIC and BIC can aid in determining which model is more suitable. According to Table 1, the *p*-value of the likelihood ratio test was significantly less than 0.05. The AIC and BIC values of the Gumbel model were both larger than those of the GEV model. Thus, the shape parameter (ξ) was not zero, and the GEV model was considered to be more adequate than the Gumbel model.

**Figure 3:**
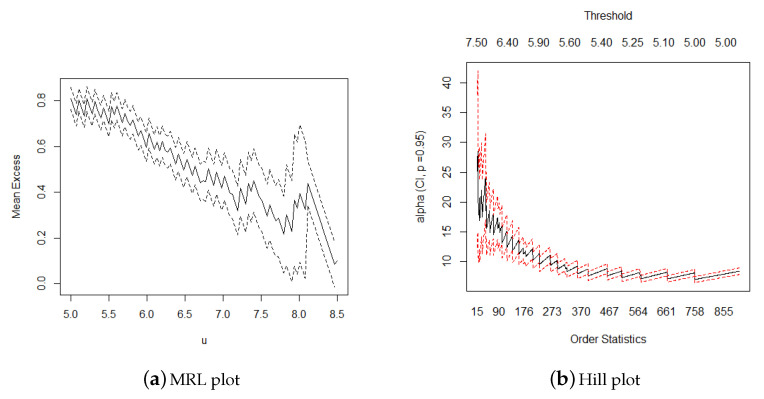
(**a**) If the mean excess begins to change linearly from a certain value, this value can be determined as a reasonable threshold. The upper and lower dotted lines represent the 95% confidence interval of the estimated value of the mean excess. (**b**) The starting point corresponding to the abscissa that causes the tail index estimator (*α*) to begin to exhibit a steady trend can be selected as the threshold.

The POT method was also applied to construct models for the earthquake magnitude of the entire Mainland China. As the POT method requires a threshold, graphical diagnostics were used to create the threshold selection visually. The MRL plot and Hill plot were used for the threshold selection. Figure 3 presents the 95% confidence interval and parameter estimation of the mean excesses and shape parameter, according to which it appeared reasonable to consider a threshold between 5.5 and 6.5.

**Figure 4:**
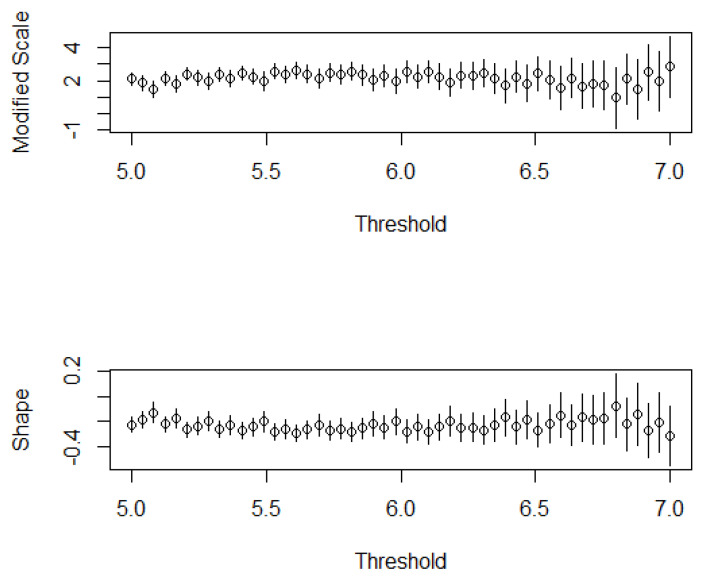
Variations in shape and scale parameters according to threshold and excesses. The length of the vertical line represents the length of the 95% confidence interval.

To select the threshold more precisely, the shape parameter and scale parameter estimation of the GP model were compared under different thresholds (Figure 4). When the threshold was 6.20, the estimated values of the two parameters were relatively stable; thus, the threshold was selected as 6.20.

The parameter estimation results of the POT models with a threshold of 6.20 are summarized in Table 2. The shape parameter of the GP model was less than zero and its standard error estimate was small. The AIC and BIC values of the GP model were smaller than those of the exponential model, and the *p*-value of the likelihood ratio test was less than 0.05 to reject the null hypothesis, thereby indicating that the GP model was more suitable.

The shape parameters of the GEV and GP models were both significantly non-zero. These were both consistent with the theory [71]: if and only if, as μ increases, the distribution of the threshold μ of the excesses uniformly converges to the GP distribution, and the BM distribution converges to the GEV distribution with the shape parameter ξ.

The right-truncated POT model described in Section 2.2.3 was used to fit the magnitude in Mainland China. The threshold was set to 6.20, which was consistent with the POT models. Table 3 summarizes that the estimated value of ξ in the right-truncated POT model (ξ≠0) tended to 0, and its log-likelihood was close to that of the right-truncated POT model with ξ = 0, indicating that the magnitudes of Mainland China were more suitable for the right-truncated POT model with ξ = 0. As the *p*-value of the likelihood ratio test was greater than 0.05, ξ was not a required parameter. Under the AIC and BIC criteria, the right-truncated POT model (ξ = 0) was the optimal model.

For comparison, the right-truncated GR distribution described in Section 2.1 was used to fit the magnitude data from Mainland China, and $t_{m}$ = 6.20 was set to be consistent with the threshold selected by the POT model. The estimated value of the parameter obtained was $\hat{\beta }$ = 1.49 and the estimated value of the right truncation point was ${\hat{T}}_{m}$ = 8.73. In this study, the GR model was used as a benchmark to compare the empirical analysis of statistical models of extreme values.

Table 4 summarizes the return level and its 95% bootstrap confidence interval for each model obtained in this section. For the BM, POT, and right-truncated POT methods, we present the return levels of the relatively optimal models, including the GEV model, GP model, and right-truncated POT model with ξ = 0. By using the return levels of the right-truncated GR model as the benchmark, we could determine the estimated accuracy of the return level for each statistical model of extreme values.

The GEV model provided the relatively lowest estimates of the recurrence levels for the two-year and five-year periods, indicating that the model was the most conservative in the estimation of the lower return levels. In contrast, the GP model provided the relatively lowest estimates of the recurrence levels in the 20-year and 50-year periods, demonstrating that the model was the most conservative in the estimation of higher return levels. Both the GEV and GP models belong to classical extreme value theory. These two models have relatively low return level estimates, which means that the classical extreme value theory may provide a relatively lower magnitude return level estimation.

The estimation of the return levels and right truncation point of the right-truncated POT model with ξ = 0 was very similar to that of the right-truncated GR model, which indicates that, in the range of statistical models of extreme values, the right-truncated POT model may be the closest to the right-truncated GR model thus far.

The return levels were compared with the magnitudes of famous earthquakes in Chinese history to obtain the return periods of earthquakes within Mainland China. The major historical earthquakes in Mainland China from 1920 to 2020 are listed in Table 5. Each earthquake in the table resulted in significant casualties and economic losses in Mainland China. We found that the 20-year return level was approximately 8.0, which means that seismic events that are similar to the earthquake that occurred in Wenchuan, Sichuan, on 12 May, 2008, with a magnitude of 8.0 Mw, have a return period of approximately 20 years in Mainland China. Furthermore, the probability of such a catastrophic earthquake in any year is 1/20. Earthquakes of 8.3 and above have a return period of approximately 50 years. Similar to the earthquake with a magnitude of 8.6 Mw that occurred in Medog, Tibet, on 15 August, 1950, the return period of Mainland China is more than 50 years. The estimated right truncation point of the earthquake magnitude distribution is approximately 8.73, which has never occurred in Mainland China. This serves as a reminder that we must consider how to avoid losses and protect life and property under such a large earthquake risk in future risk management.

The comparison of the estimated results of the above six statistical models of extreme values based on the return level of the right-truncated GR model revealed that the results of the right-truncated POT model (ξ = 0) were the closest. A model from classical extreme value theory may provide a relatively lower return level estimation of the magnitude. Therefore, when estimating the largest possible magnitude for Mainland China, the right-truncated POT model (ξ = 0) is the closest theoretical model to the geographical-based right-truncated GR model.

### 3.3. Application of Parameter Models for Earthquake Magnitude to Mainland China with Clustering

Earthquakes in Mainland China can be classified based on their similarity; that is, a certain number of categories can be obtained through clustering according to the measured variables of the earthquake, following which each type of earthquake can be modeled and analyzed. The K-means clustering method is the most commonly used approach owing to its simplicity and efficiency. In K-means clustering, the number of clusters is provided prior to calculation and each cluster is represented by an average (or a weighted average) of the centroid. The characteristic of each cluster is determined by its centroid, which is located at the center of the elements that constitute the cluster.

We first used K-means clustering to divide the seismic data of Mainland China into multiple clusters and subsequently applied the parametric model in Section 2 to each region. Similar to the analysis process in Section 3.2, after comparing the AIC and BIC values, and the *p*-value of the likelihood ratio test to obtain the optimal model for each statistical model of extreme values, the return levels of each cluster were calculated. The optimal statistical models of extreme values and the estimated value of the return level of each region were obtained by comparison with the right-truncated GR model.

#### 3.3.1. Seismic Zoning in Mainland China

**Figure 5:**
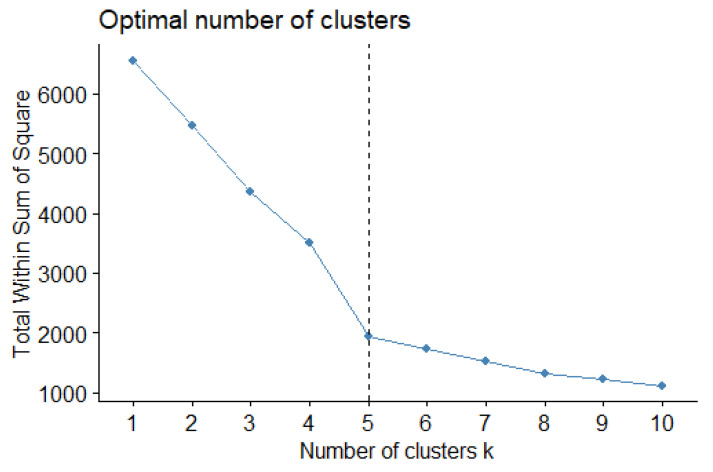
Number of clusters and total within sum of squares.

Four variables were considered in the clustering: the earthquake magnitude, depth (km), and location longitude and latitude. After standardizing the four variables, clustering was performed and Mainland China was divided into several zones according to the clustering results. Initially, five clusters were established. As illustrated in Figure 5, when K=5, the variability within each group began to stabilize. From the point of K=5, the variability within each group decreased smoothly.

Table 6 displays the standardized values of each variable in the five groups that were formulated by clustering. The second group was characterized by larger average magnitudes compared to the other groups. The characteristics of the third group were substantially deeper average seismic depths and larger average magnitudes. However, the second group was scattered irregularly throughout Mainland China and the third group had only 11 records; thus, these two groups could not be regarded as clusters.

The remaining groups (groups 1, 4, and 5 in Table 6) had variables that were close to the origin. The earthquakes of these groups were scattered on the map of Mainland China, covering most areas. As the ideal situation was to create regions that could cover a large geographical area, three clusters were considered. The third group was merged into the fifth group because the 11 records of the third group were all at the border of Jilin Province and were within the geographical scope of the fifth group. The records of the third group were separated during clustering owing to the immense seismic depth. As the geographical distribution of the second group was excessively scattered, independent modeling was of little significance. The second group was not removed but rather incorporated into the other groups using K=3 clustering.

**Figure 6:**
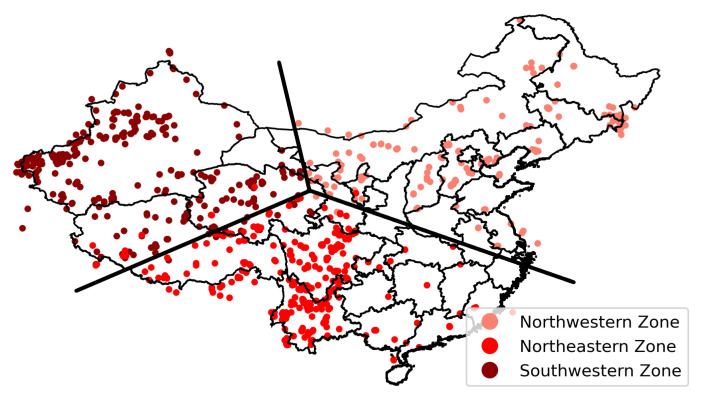
Seismic zoning in Mainland China. The different colors on the map represent different zones, and each point on the map represents the geographical location of each earthquake event from 1920 to 2020.

The seismic zoning results of Mainland China are depicted in Figure 6. The seismic zoning in this case was based on the characteristics of earthquakes and was obtained by clustering in the field of statistics, which differs from the zoning obtained by geological features and plate movement [72,73]. The northwestern zone covers the entire Xinjiang Uygur Autonomous Region, the northern part of the Tibet Autonomous Region, and Qinghai. The northeastern zone is located to the East of the Yellow River, including Shanxi, the Beijing–Tianjin–Hebei area, Inner Mongolia, and the three northeastern provinces. The southwestern zone comprises the south of the Yellow River, including Sichuan, Chongqing, Yunnan, and Guizhou, as well as parts of Qinghai and Tibet.

After dividing Mainland China into three zones, each zone was modeled using the parametric models outlined in Section 2.

#### 3.3.2. Estimation Results for Northwestern Zone

**Figure 7:**
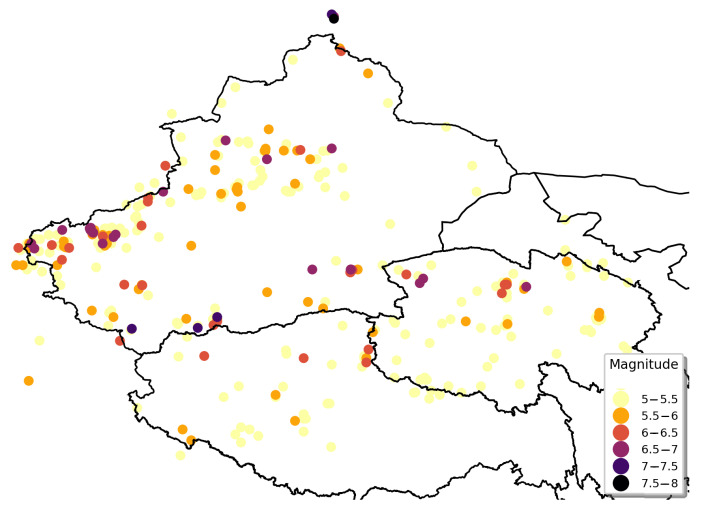
Geographical distribution of seismic records in northwestern zone.

The geographical distribution of the seismic records in the northwestern zone is presented in Figure 7. Many of the earthquakes in the northwestern zone had magnitudes of 5 to 5.5 Mw and most of the earthquakes with magnitudes above 6 Mw were located on the northwest border of Xinjiang, the southern border of Xinjiang, and the northern border of Qinghai Province. There were many historical earthquakes with magnitudes of 5 Mw and above in the northwestern zone, but the possibility of severe earthquakes was low.

Table 7 and Table 8 display the estimation results of the statistical models of extreme values. According to the AIC, BIC, and likelihood ratio test *p*-value, the Gumbel and GP models were optimal. Using the MRL plot and Hill plot, the threshold of the GP model was selected as 5.75. When fitting the right-truncated POT model, the threshold was consistent with the POT model. The parameter estimation results in Table 8 demonstrate that the parameter estimation of ξ tended to 0 and the log-likelihood values of the two models were very close, indicating that the POT model with right truncation and ξ = 0 was a more suitable model. Based on the *p*-value of the likelihood ratio test, AIC, and BIC, the right-truncated POT model (ξ = 0) was the optimal model. When fitting the right-truncated GR model, $t_{m}=5.75$ was set, which was consistent with the POT model. The estimated parameter value obtained was $\hat{\beta }$ = 1.64, and the estimated value of the right truncation point was ${\hat{T}}_{m}=8.13$.

The return levels of the parametric models for earthquake magnitude are listed in Table 9. The return levels in this area were less than those estimated by the records of the entire Mainland China. For example, a return period of approximately 50 years was observed for an earthquake with a magnitude of 7.8 in the northwestern zone, whereas the return level of 7.8 was less than 20 years for Mainland China. When the return level of the right-truncated GR model was used as the benchmark, the results of the right-truncated POT model (ξ = 0) were the closest, which also had the lowest AIC and BIC values among the statistical models of extreme values.

#### 3.3.3. Estimation Results for Northeastern Zone

**Figure 8:**
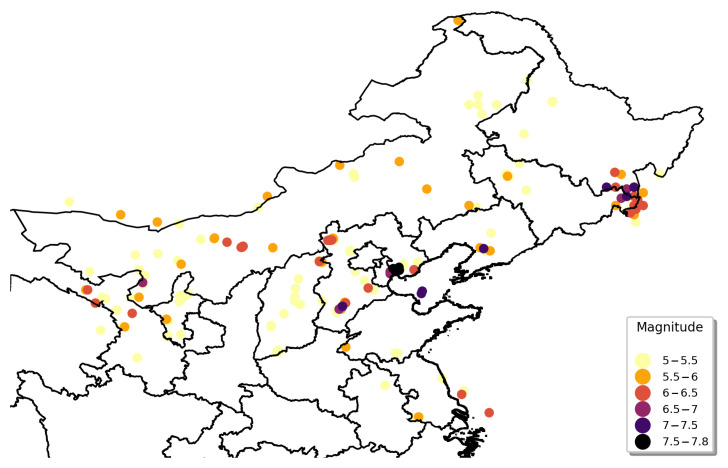
Geographical distribution of seismic records in northeastern zone.

The geographical distribution of earthquakes in the northeastern zone is illustrated in Figure 8. Earthquakes in the northeastern zone were concentrated on the border of Jilin and Heilongjiang, which were characterized by a high frequency of earthquake events, large magnitudes, and nearness of geographical locations. Several severe earthquakes were also located in the Beijing–Tianjin–Hebei region. Shanxi and Inner Mongolia had a certain amount of moderate earthquakes, whereas other areas such as Shaanxi Province had no historical earthquakes above 5 Mw.

The estimation results of the BM and POT models of extreme values are summarized in Table 10. According to the AIC, BIC, and likelihood ratio test *p*-value, the Gumbel and GP models were optimal. The threshold of the GP model was selected as 5.40, with which the corresponding parameters of the right-truncated POT model and right-truncated GR model were consistent. The estimation results of the right-truncated POT model are summarized in Table 11, which demonstrate that the parameter estimation of ξ tended to 0 and the log-likelihood values of the two models were very close, indicating that the POT model with right truncation and ξ = 0 was more suitable. When fitting the right-truncated GR model, $t_{m}$ = 5.40 was set, the estimated parameter value obtained was $\hat{\beta }$ = 1.26, and the estimated value of the right truncation point was $\hat{T_{m}}=7.95$.

The return levels and their 95% bootstrap confidence intervals of the parametric models for earthquake magnitudes are summarized in Table 12. The probability of large earthquakes in this region was low and even smaller than that of the northwestern zone. The two-year return level was lower than 6, indicating that most of the earthquakes in the northeastern zone and were moderate earthquakes, as defined in Section 3.1. When using the return level of the right-truncated GR model as the benchmark, the results of the right-truncated POT model (ξ = 0) remained the closest.

#### 3.3.4. Estimation Results for Southwestern Zone

**Figure 9:**
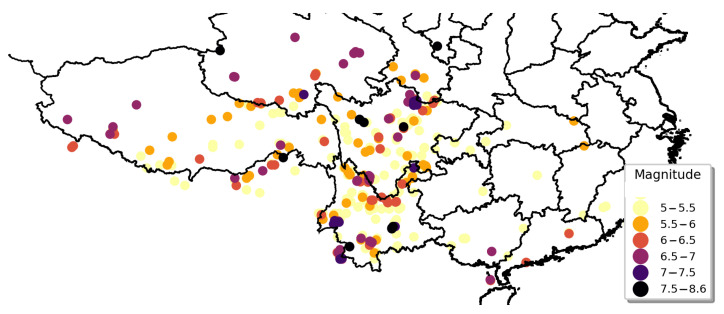
Geographical distribution of seismic records in southwestern zone.

The geographical distribution of earthquakes in the southwestern zone is depicted in Figure 9. Compared with the northwestern and northeastern zones, the density of earthquake events was significantly more intensive and the magnitudes were generally higher. Earthquakes in the southwestern zone were concentrated in Sichuan, Yunnan, and the boundaries of Qinghai Province. In the south of Sichuan and Yunnan, fewer earthquake events were observed and their magnitudes were low.

The estimation results of the BM and POT models are summarized in Table 13. According to the AIC, BIC, and likelihood ratio test *p*-value, the optimal models for the southwestern zone were the Gumbel and GP models, which was the case for the other two zones. The right-truncated POT model was used for fitting and the parameters obtained are displayed in Table 14. Table 14 summarizes that the estimated value of ξ in the right-truncated POT model (ξ≠ 0) tended to 0 and the log-likelihood value was consistent with that of the right-truncated POT model with ξ = 0, indicating that the POT model with right truncation and ξ = 0 was more suitable for earthquakes in the southwestern zone. When comparing the model with traditional extreme value models (the Gumbel and GP models), the right-truncated POT model (ξ = 0) was the optimal model under the AIC and BIC criteria. The right-truncated GR model was also applied to earthquakes in the southwestern zone and $t_{m}$ = 6.00 was set, which was consistent with the threshold selected by the POT model. The estimated parameter was $\hat{\beta }$ = 1.21 and the estimated value of the right truncation point was ${\hat{T}}_{m}$ = 8.76, which was slightly higher than that of the estimation result in Mainland China.

The calculated return levels and their 95% bootstrap confidence intervals are summarized in Table 15. When the return level of the right-truncated GR model was used as the benchmark, the results of the right-truncated POT model (ξ = 0) were the closest among the statistical models of extreme values. The probability of large earthquakes in this region was relatively high, and the two-year return level reached 6.5. The estimation results were quite similar to the return levels in Section 3.2 and the southwestern zone. The high-level earthquakes of Mainland China originated from the southwestern zone; thus, Chinese earthquake risk management should focus on the southwestern zone.

### 3.4. Discussion of Return Period Estimation

In this section, we discuss the results of the return period estimation in Section 3.2 and Section 3.3. In this study, the right-truncated GR model was used as a benchmark to compare three types of statistical models of extreme values. Therefore, we compared the statistical models of extreme values obtained in Section 3.2 and Section 3.3 with the truncated GR model using the QQ plots of the right tail quantile to demonstrate the differences in the return level estimation of the models more clearly. We plotted the quantiles for Mainland China and the three zones separated by K-means clustering according to the corresponding parameter models. The 40% to 99% quantile of each parameter model was selected to draw the QQ plot and a step size of 1 was used for the quantile value.

**Figure 10:**
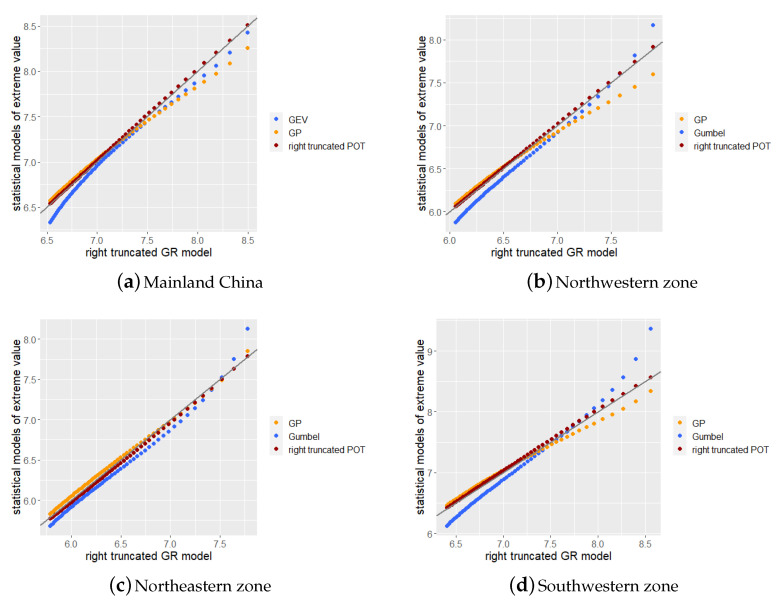
QQ plots of right tail to compare differences between statistical models of extreme values and right-truncated GR model. Each dot represents the same quantile in the statistical model of extreme values and the right-truncated GR model.

As can be observed from Figure 10, among the four QQ plots, the right-truncated POT model exhibited the best performance, demonstrating the highest fitness to the right-truncated GR model. The Gumbel model had a relatively low value in the lower part of the plot and a relatively high value in the higher part. As noted in Section 3.3, the return levels for two years and five years of the Gumbel model were almost the lowest, whereas the return levels for 20 years and 50 years were almost the highest. As illustrated in Figure 10a,c,d, the estimated return level of the GP model was lower at the high quantile, indicating that the estimation may be lower than the actual situation when using the GP model to estimate the return level of a high return period.

The horizontal and vertical axes of Figure 10b–d can be used to compare the estimated return levels of each zone. The southwestern zone had a higher return level, whereas the northwestern and northeastern zones had lower return levels under the same return period. This demonstrates that there were significant differences in the occurrence of earthquakes among the zones, and it will be meaningful to conduct a regional discussion on earthquakes in China.

Moreover, the methodology in this study did not consider the locations of faults or tectonic plates.

## 4. Conclusions

Statistical models of extreme values can provide earthquake magnitude distributions from a data-driven perspective and provide model construction that differs from that according to the earthquake occurrence mechanism in seismology. This study used three types of statistical models of extreme values to estimate the return periods of the largest possible earthquake magnitudes. The right-truncated GR model was used as a benchmark to compare the estimation results. Based on the geographical features of earthquake events, we used K-means clustering to provide scientific earthquake zoning for Mainland China and we summarized the earthquake characteristics in each zone. In an attempt to establish several geographical characteristics of earthquakes, we used the four variables mentioned in Section 3.3.1 to conduct K-means clustering.

The analysis was carried out with and without clustering of earthquakes in Mainland China. K-means clustering was used to divide Mainland China into three zones: the northwestern zone, northeastern zone, and southwestern zone. The occurrence frequency of large earthquakes in the northwestern and southwestern zone was low, resulting in fewer catastrophic losses. The occurrence frequency of earthquakes in the southwestern zone was high and the occurrence rate of large earthquakes was higher than that in the two other regions, which means that there were more earthquake events causing significant catastrophe losses. The southwestern zone includes Sichuan, Yunnan, part of the Tibet Autonomous Region, and Guizhou, which are high earthquake-prone areas. According to the analysis of each region using the models mentioned in Section 2, the right-truncated POT model was the optimal statistical model. When comparing the estimations of the return levels of each zone, the estimation results of the southwestern zone were quite similar to those of Mainland China. High-level earthquakes in Mainland China almost always originated from the southwestern zone; thus, Chinese earthquake risk management should focus on the southwestern zone. Compared with the estimation results of the return level of the model with the right-truncated effect, the return level provided by the right-truncated GR model based on a geophysical perspective was very close to the generalized Pareto distribution. We concluded that the right-truncated POT model was relatively optimal when estimating the return level, which could perform better than classical extreme value theorem models. The three zones of Mainland China obtained through K-means clustering have different earthquake magnitude characteristics. The differences among the three zones should be considered. The differences in the estimation results of the return periods in the three zones indicate that seismic hazard prevention and management in should be adapted to the local conditions of specific areas.

From a modeling perspective, probability distributions can provide a data-driven distribution of the earthquake magnitude. A risk prediction model of earthquake catastrophes can be obtained based on statistics, and the risk of future earthquakes can be quantified probabilistically. From a practical perspective, the statistical probability model based on earthquakes does not require abundant geographical observation data. This method can replace the seismological model for areas with a lack of geographical data and observation conditions, to provide effective earthquake magnitude model estimation and future earthquake risk prediction. This paper has presented an accurate, economical, and efficient solution for the construction of earthquake magnitude models. From an application perspective, if the three zones are modeled separately, more accurate results can be obtained and risk management can be conducted more effectively.

However, several limitations should be noted. First, owing to the incomplete historical data records, only earthquakes between 1920 and 2020 with magnitudes of 5 Mw and above were considered. If data with a larger time span and a more complete earthquake catalog can be obtained, the estimated results will be more reliable. Second, earthquake magnitude modeling has not been studied in conjunction with seismology and the plate motion of earthquakes; thus, an interdisciplinary study that includes geology should be considered. Third, only the magnitude, longitude, latitude, and depth of earthquakes were used in the clustering. Future research should incorporate additional earthquake-related geological variables into the cluster analysis.

## Figures and Tables

**Table 1:** Parameter estimation of block maxima models for Mainland China.

	GEV Model			Gumbel Model	
	μ	σ	ξ	μ	σ
Estimated parameters	6.28	0.70	−0.19	6.21	0.68
Standard error estimates	0.08	0.06	0.07	0.08	0.05
AIC	203.32			207.34	
BIC	209.65			211.69	
*p*-value				0.01	

**Table 2:** Parameter estimation of POT models for Mainland China.

	GP Model		Exponential Model
	σ	ξ	σ
Estimated parameters	0.79	−0.28	0.62
Standard error estimates	0.07	0.05	0.05
AIC	177.95		189.88
BIC	189.49		193.06
*p*-value			2.0 × 10${}^{-4}$

**Table 3:** Parameter estimation of the right truncated POT models for Mainland China.

Parameter Estimation	Right Truncated POT Model (ξ≠0)	Right Truncated POT Model (ξ=0)
σ	0.69	0.69
ξ	−8.5 × 10${}^{-3}$	
$T_{m}$	8.74	8.73
Log likelihood	−86.04	−86.16
AIC	176.08	174.33
BIC	182.44	177.51
*p*-value	0.62	

**Table 4:** Return level and its 95% bootstrap confidence interval of parametric models for the earthquake magnitude in Mainland China.

Return Period	GEV Model	GP Model	Right Truncated POT Model (ξ = 0)	Right Truncated GR Model
2 years	6.53 (6.36, 6.68)	6.70 (6.62, 6.76)	6.66 (6.60, 6.73)	6.65 (6.59, 6.70)
5 years	7.19 (7.01, 7.39)	7.23 (7.11, 7.34)	7.24 (7.13, 7.38)	7.22 (7.11, 7.34)
20 years	7.87 (7.60, 8.17)	7.81 (7.65, 8.00)	8.00 (7.85, 8.24)	7.97 (7.81, 8.21)
50 years	8.21 (7.86, 8.60)	8.09 (7.88, 8.35)	8.34 (8.15, 8.69)	8.32 (8.12, 8.65)

**Table 5:** Historical major earthquakes in Mainland China (Mw ≥ 7.9).

Time	Location	Mw
15 Augest 1950	Medog, Tibet	8.6
16 December 1920	Haiyuan, Gansu	8.5
14 November 2001	The junction of Xinjiang Uygur Autonomous Region, Qinghai Province and Tibet Autonomous Region	8.1
12 May 2008	Wenchuan, Sichuan	8.0
6 Febrary 1973	Luhuo, Sichuan	7.9
23 May 1927	Gulang, Gansu	7.9
27 September 2003	The junction of Russia, Mongolia, and China	7.9

**Table 6:** Standardized variable characteristics of clustering.

Group	Standardized Longitude	Standardized Latitude	Standardized Magnitude	Standardized Depth
1	−1.21	0.83	−0.11	−0.07
2 *	0.14	−0.67	1.62	−0.11
3 **	2.34	1.31	0.65	8.85
4	0.02	−0.69	−0.55	−0.13
5	1.26	0.81	−0.23	−0.13

* Incorporated into the group 1, 4 and 5. ** Merged into the group 5.

**Table 7:** Estimation results of the statistical models of extreme value for Northwestern zone.

	**GEV Model**			**Gumbel Model**	
	***μ***	***σ***	***ξ***	***μ***	***σ***
Estimated parameters	5.87	0.53	−0.11	5.83	0.51
Standard error estimates	0.08	0.06	0.10	0.07	0.05
AIC	104.21			103.12	
BIC	108.78			106.18	
*p*-value				0.34	
	**GP Model**			**Exponential Model**	
	***μ***	***σ***	***ξ***	***μ***	***σ***
Estimated parameters	5.75	0.73	−0.29	5.75	0.57
Standard error estimates	/	0.09	0.07	/	0.06
AIC	76.32			82.96	
BIC	85.89			85.48	
*p*-value				3.3 × 10${}^{-3}$	

**Table 8:** Parameter estimation of right truncated POT model for Northwestern zone.

	Right Truncated POT Model (ξ≠ 0)	Right Truncated POT Model (ξ = 0)
σ	0.62	0.63
ξ	−5.5 × 10${}^{-3}$	
$T_{m}$	8.16	8.15
Log likelihood	−34.85	−34.90
AIC	73.70	71.81
BIC	78.75	74.33
*p*-value	0.75	

**Table 9:** Return level and its 95% bootstrap confidence interval of parametric models for the earthquake magnitude in Northwestern zone.

Return Period	Gumbel Model	GP Model	Right Truncated POT Model (ξ = 0)	Right Truncated GR Model
2 years	6.02 (5.86, 6.18)	6.21 (6.10, 6.29)	6.17 (6.09, 6.25)	6.16 (6.09, 6.22)
5 years	6.60 (6.38, 6.82)	6.69 (6.54, 6.83)	6.70 (6.58, 6.89)	6.69 (6.56, 6.83)
20 years	7.34 (7.04, 7.68)	7.21 (7.00, 7.49)	7.41 (7.21, 7.86)	7.38 (7.22, 7.67)
50 years	7.82 (7.44, 8.23)	7.45 (7.18, 7.86)	7.75 (7.46, 8.43)	7.72 (7.52, 8.14)

**Table 10:** Estimation results of the statistical models of extreme values for Northeastern zone.

	**GEV Model**			**Gumbel Model**	
	***μ***	***σ***	***ξ***	***μ***	***σ***
Estimated parameters	5.64	0.54	−4.0 × 10${}^{-3}$	5.64	0.54
Standard error estimates	0.08	0.06	0.12	0.07	0.05
AIC	143.97			141.97	
BIC	149.25			145.49	
*p*-value				0.97	
	**GP Model**			**Exponential Model**	
	***μ***	***σ***	***ξ***	***μ***	***σ***
Estimated parameters	5.40	0.897	−0.25	5.40	0.71
Standard error estimates	/	0.13	0.10	/	0.07
AIC	142.37			144.78	
BIC	152.42			147.46	
*p*-value				0.04	

**Table 11:** Parameter estimation of right truncated POT model for Northeastern zone.

	Right Truncated POT Model (ξ≠ 0)	Right Truncated POT Model (ξ = 0)
σ	0.83	0.76
ξ	−0.19	
$T_{m}$	8.14	7.98
Log likelihood	−57.71	−57.76
AIC	119.43	117.53
BIC	124.79	120.21
*p*-value	0.76	

**Table 12:** Return level and its 95% bootstrap confidence interval of parametric models for the earthquake magnitude in Northeastern zone.

Return Period	Gumbel Model	GP Model	Right Truncated POT Model (ξ = 0)	Right Truncated GR Model
2 years	5.83 (5.68, 6.00)	5.97 (5.84, 6.10)	5.90 (5.80, 6.90)	5.92 (5.83, 6.00)
5 years	6.45 (6.22, 6.68)	6.59 (6.38, 6.80)	6.52 (6.34, 6.72)	6.56 (6.40, 6.73)
20 years	7.25 (6.92, 7.60)	7.29 (7.08, 7.52)	7.30 (7.12, 7.51)	7.33 (7.18, 7.50)
50 years	7.75 (7.36, 8.20)	7.64 (7.40, 7.92)	7.63 (7.52, 7.78)	7.64 (7.54, 7.77)

**Table 13:** Estimation results of the statistical models of extreme values for Southwestern zone.

	**GEV Model**			**Gumbel Model**	
	***μ***	***σ***	***ξ***	***μ***	***σ***
Estimated parameters	6.12	0.74	−0.14	6.06	0.72
Standard error estimates	0.11	0.07	0.09	0.01	0.07
AIC	151.98			152.10	
BIC	157.05			155.48	
*p*-value				0.15	
	**GP Model**			**Exponential Model**	
	***μ***	***σ***	***ξ***	***μ***	***σ***
Estimated parameters	6.00	0.98	−0.33	6.00	0.75
Standard error estimates	/	0.11	0.07	/	0.07
AIC	162.77			173.45	
BIC	173.16			176.24	
*p*-value				4 × 10${}^{-4}$	

**Table 14:** Parameter estimation of right truncated POT model for Southwestern zone.

	Right Truncated POT Model (ξ≠ 0)	Right Truncated POT Model (ξ = 0)
σ	0.82	0.87
ξ	−8.6 × 10${}^{-3}$	
$T_{m}$	8.75	8.75
Log likelihood	−77.99	−78.01
AIC	159.98	158.01
BIC	165.57	160.81
*p*-value	0.85	

**Table 15:** Return level and its 95% bootstrap confidence interval of parametric models for the earthquake magnitude in Southeastern zone.

Return Period	Gumbel Model	GP Model	Right Truncated POT Model (ξ = 0)	Right Truncated GR Model
2 years	6.33 (6.12, 6.53)	6.61 (6.51, 6.71)	6.57 (6.55, 6.60)	6.54 (6.47, 6.62)
5 years	7.14 (6.89, 7.46)	7.23 (7.08, 7.40)	7.26 (7.20, 7.37)	7.22 (7.08, 7.36)
20 years	8.20 (7.84, 8.66)	7.88 (7.66, 8.12)	8.09 (7.92, 8.38)	8.05 (7.90, 8.26)
50 years	8.87 (8.42, 9.492)	8.17 (7.86, 8.44)	8.42 (8.20, 8.80)	8.40 (8.27, 8.63)

## Data Availability

The dataset was provided by China Earthquake Networks Center, National Earthquake Data Center (http://data.earthquake.cn). Accessed on 9 September 2020.

## Abbreviations

The following abbreviations are used in this manuscript:

PSHA	Probabilistic seismic hazard analysis
POT	Peak over threshold
GP	Generalized Pareto
GR	Gutenberg–Richter
AIC	Akaike Information Criterion
BIC	Bayesian Information Criterion
GEV	Generalized Extreme Value
MRL	Mean Residual Life
